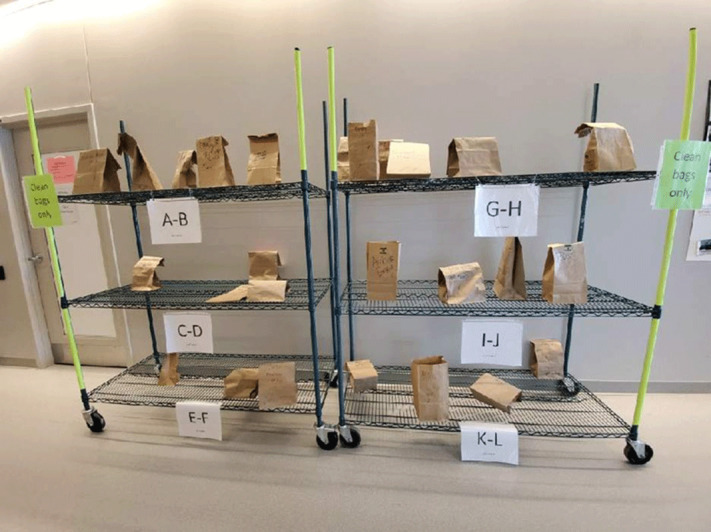# Using Low-Heat Decontamination to Allow N95 and PPE Reuse During the COVID-19 Pandemic

**DOI:** 10.1017/ash.2021.118

**Published:** 2021-07-29

**Authors:** Amy Kressel, Katie Swafford, DJ Shannon, Rachel Cathey, Jamie R. Fryar, Matthew E. Royal, Ryan Noyes

## Abstract

**Background:** US healthcare facilities experienced significant personal protective equipment (PPE) shortages, including N95 masks, in the spring and summer of 2020. The Centers for Disease Control and Prevention issued guidance for extended use, reprocessing, and reuse of N95s. Eskenazi Health (EH) implemented a program to reprocess N95s and other PPE on-site using low-heat decontamination (LHD). EH considered large-scale and small-scale ultraviolet (UV), hydrogen peroxide vapor, and LHD for on-site reprocessing of N95s. All of these methods allowed up to 3 reprocessing cycles according to most literature available at the time. However, each method differed in feasibility and acceptability to staff. EH chose to implement LHD based on both considerations. **Methods:** Numerous small-group meetings were held in April 2020 to determine the feasibility and acceptability of N95 reprocessing methods. Staff wanted a method that was easy for the end user, had quick turnaround, and allowed them to retrieve their own N95s. They favored a method that could be used for all PPE. EH had deployed numerous small UV machines that individuals could use for N95s. The UV machines could not be scaled up easily. To scale up, a multidisciplinary team comprising infection prevention, biomedical engineering, and sterile processing representatives reviewed available methods and implemented LHD. Biomedical engineers determined that existing blanket warmers could be reprogrammed and repurposed for low-heat decontamination. Food warmers were also available but were not needed. Biomedical engineers reprogrammed the blanket warmers to 70°C and developed a wicking system using a towel and water tray to maintain humidity; decontamination took 30 minutes. Testing runs determined that both N95s and eye protection tolerated LHD without apparent damage. Infection prevention staff developed a workflow in which staff deposited all PPE in a paper bag; the PPE bag was centrally reprocessed, marked (Figure 1), and returned to designated locations (Figure 2) for staff to retrieve their original PPE. Sterile processing staff facilitated the reprocessing workflow, and elective surgeries were canceled during the COVID-19 surge. **Results:** From April 20, 2020, to July 19, 2020, 7,512 units were decontaminated with LHD. If each N95 was sterilized thrice (4 uses per N95), then LHD reduced the need to purchase 22,536 N95s. Restarting elective surgeries decreased staff and support from sterile processing; the space was needed for other purposes; and N95 availability increased. All of these factors led to the discontinuation of LHD. **Conclusions:** LHD enables reprocessing of N95s and other PPE using existing assets. LHD is advantageous because of scalability and the capacity to provide staff with their own reprocessed PPE.

**Funding:** No

**Disclosures:** None

**Figure 1.**
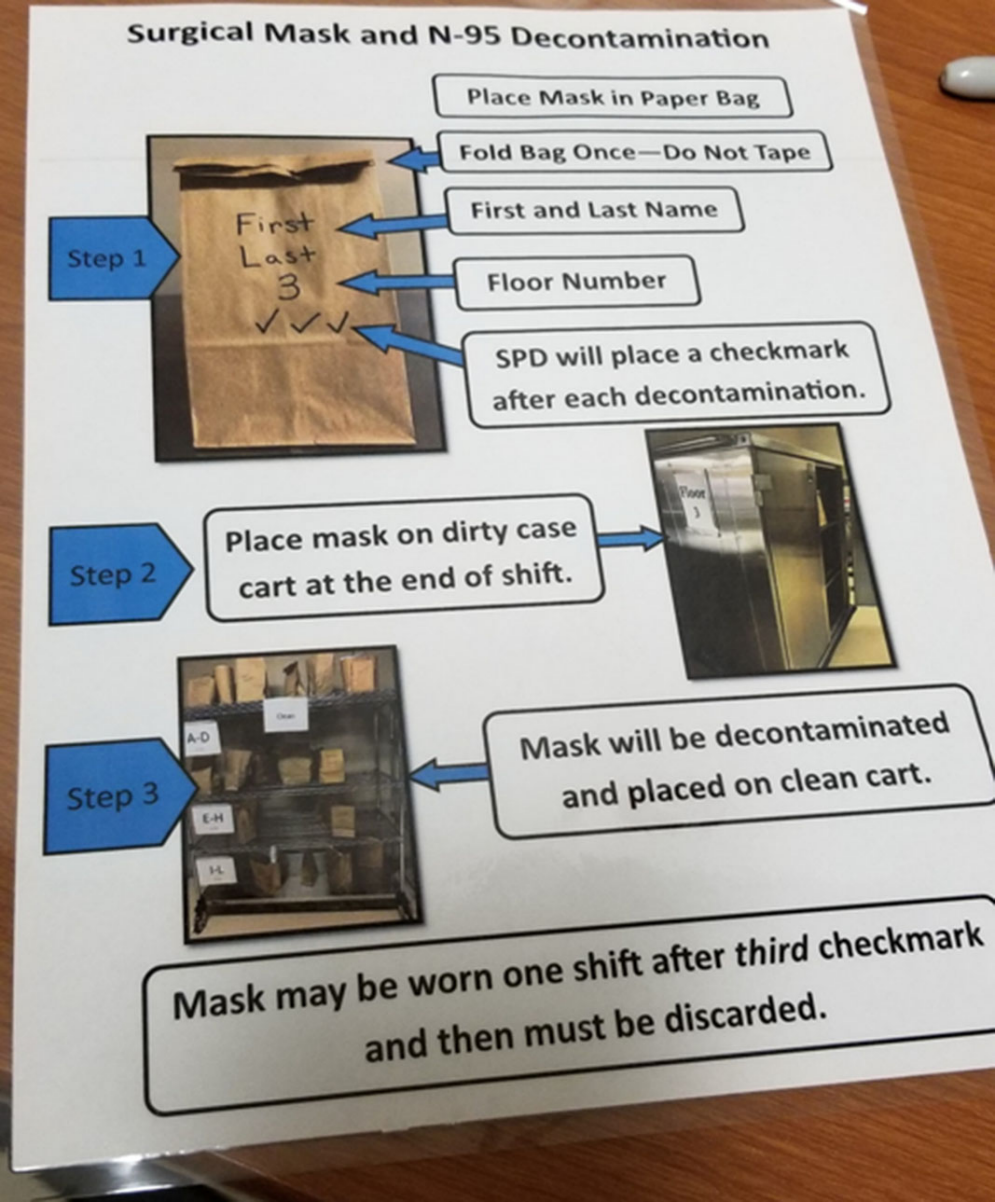

**Figure 2.**